# Coordinated Degradation of Replisome Components Ensures Genome Stability upon Replication Stress in the Absence of the Replication Fork Protection Complex

**DOI:** 10.1371/journal.pgen.1003213

**Published:** 2013-01-17

**Authors:** Laura C. Roseaulin, Chiaki Noguchi, Esteban Martinez, Melissa A. Ziegler, Takashi Toda, Eishi Noguchi

**Affiliations:** 1 Department of Biochemistry and Molecular Biology, Drexel University College of Medicine, Philadelphia, Pennsylvania, United States of America; 2 Laboratory of Cell Regulation, Cancer Research UK, London Research Institute, Lincoln's Inn Field Laboratories, London, United Kingdom; University of Massachusetts Medical School, United States of America

## Abstract

The stabilization of the replisome complex is essential in order to achieve highly processive DNA replication and preserve genomic integrity. Conversely, it would also be advantageous for the cell to abrogate replisome functions to prevent inappropriate replication when fork progression is adversely perturbed. However, such mechanisms remain elusive. Here we report that replicative DNA polymerases and helicases, the major components of the replisome, are degraded in concert in the absence of Swi1, a subunit of the replication fork protection complex. In sharp contrast, ORC and PCNA, which are also required for DNA replication, were stably maintained. We demonstrate that this degradation of DNA polymerases and helicases is dependent on the ubiquitin-proteasome system, in which the SCF^Pof3^ ubiquitin ligase is involved. Consistently, we show that Pof3 interacts with DNA polymerase ε. Remarkably, forced accumulation of replisome components leads to abnormal DNA replication and mitotic catastrophes in the absence of Swi1. Swi1 is known to prevent fork collapse at natural replication block sites throughout the genome. Therefore, our results suggest that the cell elicits a program to degrade replisomes upon replication stress in the absence of Swi1. We also suggest that this program prevents inappropriate duplication of the genome, which in turn contributes to the preservation of genomic integrity.

## Author Summary

Replication stress interferes with the normal progression of the replication fork. Under these conditions, cells activate the replication checkpoint to coordinate DNA repair with cell cycle arrest. The current understanding is that, in response to replication block, this checkpoint stabilizes replication forks and replisome structures to achieve accurate DNA replication. However, it would also be advantageous for the cell to stop DNA replication and reorganize the replisome structures when conditions are not ideal, but such mechanisms have not been explored. In this study, we describe a mechanism that regulates replisome stability in response to replication stress. We found that replisome components become highly unstable and degraded when replication forks are perturbed in the absence of Swi1, a subunit of replication fork protection complex. We demonstrate that replisome degradation is dependent on the SCF^Pof3^ ubiquitin ligase complex. Strikingly, when we forced cells to stabilize replisome components, cells underwent abnormal DNA replication, leading to mitotic catastrophes. Thus, our study provides novel mechanistic insights into understanding how the replication machinery is regulated to achieve faithful duplication of the genome upon replication stress.

## Introduction

Initiation of DNA replication is directed by the formation of the pre-replication complex (pre-RC) at the origin of replication [1]. The pre-RC includes a number of essential replication proteins such as origin recognition complex (ORC), Cdc6, Cdt1, and the mini-chromosome maintenance (MCM) DNA helicase complex. However, to initiate actual DNA synthesis, additional factors are needed to facilitate the unwinding of origins and generation of replication forks. These factors include Cdc45, go-ichi-ni-san (GINS), replication protein A (RPA), proliferating cell nuclear antigen (PCNA), and other accessory factors prior to the loading of DNA polymerases. Together, these factors form the replisome complex at the replication fork [1]. However, how the cell maintains the integrity of the replisome is not well understood.

In response to replication stress, cells activate the DNA replication checkpoint to allow time for DNA repair. Central to this system are protein kinases such as human ATM and ATR, fission yeast Rad3, and budding yeast Mec1 [2]–[6]. These kinases are required for activation of downstream effector kinases by phosphorylation. In the fission yeast *Schizosaccharomyces pombe*, Rad3 activates Cds1 and Chk1 kinases in response to replication stress or DNA damage, facilitating DNA repair and recombination pathways [2], [4], [7]. Another essential function of the replication checkpoint is to stabilize replication forks by maintaining proper assembly of replisome components and preserving DNA structures during DNA replication problems [8]–[12]. Recent studies found that ancillary factors that are not essential for DNA synthesis but are important for DNA replication accuracy also travel with moving replication forks. Such factors include fission yeast Swi1 and Swi3, which together form the replication fork protection complex (FPC) and are required for efficient activation of the replication checkpoint kinase Cds1 and for the stabilization of replication forks [13]–[16]. In the absence of Swi1 or Swi3, cells accumulate abnormal fork structures that lead to Rad22 (the Rad52 orthologue) DNA repair foci formation and accumulation of recombination structures during S phase [16], [17]. The functions of the Swi1–Swi3 complex appear to be conserved among eukaryotes [14], [15], [18]–[20]. Studies show that Swi1–Swi3 orthologues (Tof1–Csm3 in budding yeast, and Timeless–Tipin in vertebrates) are components of the replisome, are involved in fork stabilization, and regulate the intra-S phase checkpoint [18], [21]–[28]. Furthermore, genetic studies in yeast also suggest that the Swi1–Swi3 FPC has roles in coordinating leading- and lagging-strand DNA synthesis and in coupling DNA polymerase and helicase activities at the replication fork [15], [16], [29]. However, how the FPC protects moving replication forks and coordinates with multiple genome maintenance processes at the replication fork is not well understood.

Replication checkpoint studies have typically used chemical agents to stall replication forks. However, emerging evidence indicates that there are a number of chromosome regions that present obstacles to DNA replication. These include programmed fork blocking sites, DNA-binding proteins such as the transcription machinery, and DNA secondary structures caused by repeat sequences. These sites are considered to be difficult to replicate, causing arrest of replication forks or even fork breakage [30]–[35]. Fork arrest at difficult-to-replicate genome sites can promote both genome instability and stability depending on the circumstances. For example, polar fork pausing at rDNA loci stimulates recombination-dependent rDNA repeat expansion and contraction, which can lead to rDNA instability. On the contrary, this polar fork pausing is also required to coordinate directionality of replication and transcription at rDNA loci, preventing genome instability due to head-on collisions of the replisome and transcriptional machinery [36], [37]. Interestingly, studies found that FPC-related proteins are required for a number of fork arrest events, which are mediated by DNA–protein complexes. These include fork pausing at the rDNA loci, the fission yeast mating-type locus, tRNA loci, and highly transcribed RNA polymerase II genes [14], [29], [38]–[41]. At rDNA loci, loss of FPC causes hyper recombination, leading to contraction of rDNA repeats [17], [40], [42]. Similarly, the high rate of transcription and the presence of DNA-binding factors increase the chances of the replisome colliding with a transcription fork. Indeed, studies in fission yeast revealed that Swi1 is required to prevent DNA damage and hyper recombination activity at these natural obstacles scattered throughout the genome [43]–[45].

In addition to these DNA–protein complex-mediated fork barriers, repeat DNA sequences themselves also cause genome instability in the absence of FPC-related proteins. At these sites, instead of promoting fork stalling, FPC appears to prevent or reduce the rate of fork stalling when the fork encounters DNA secondary structures caused by repeat sequences. Therefore, in the absence of FPC, fork stalling results in elevated levels of ssDNA exposed at the replication fork, which appear to cause genome instability due to expansion and contraction at DNA structure-based impediments [46]–[51]. Thus, the mechanisms of the FPC-dependent fork regulation at repeat regions and at DNA–protein complex-mediated fork barriers are different. However, accumulated evidence indicates that FPC proteins are required for smooth passage of replication forks and for suppression of replication stresses at these natural impediments [14].

Therefore, in this study, we used *swi1*Δ as a model to understand replication stress response mechanisms. Strikingly, we have found that replicative DNA polymerases and helicases are highly unstable in the absence of Swi1. Our investigation revealed that this degradation is mediated by the ubiquitin-proteasome system, in which the SCF^Pof3^ (Skp1/Cul1/F-box) ubiquitin ligase complex is involved. In the absence of Pof3, *swi1*Δ cells undergo mitotic catastrophes, suggesting the importance of proteasome-dependent replisome regulation in preserving genomic integrity. Considering that *swi1*Δ cells accumulate replication stress at difficult-to-replicate regions throughout the genome, our findings suggest that ubiquitin-dependent degradation of replisome components play a critical role in genome duplication in response to replication stresses.

It is widely understood that checkpoint proteins stabilize replication forks and replisomes in response to replication stress. However, our findings suggest an alternative mechanism that cells abrogate replisome functions when the fork encounters obstacles. Therefore, our study proves new mechanistic insights into the understanding of the replication stress response. In addition, although a number of studies have focused on the processes of replication initiation and regulation of fork progression, how the replisome itself is regulated is still largely unknown. Therefore, our findings also fill the knowledge gap in the regulation of replisome components in the DNA replication program.

## Results

### Replisome components are unstable in *swi1*Δ cells

**Figure 1:**
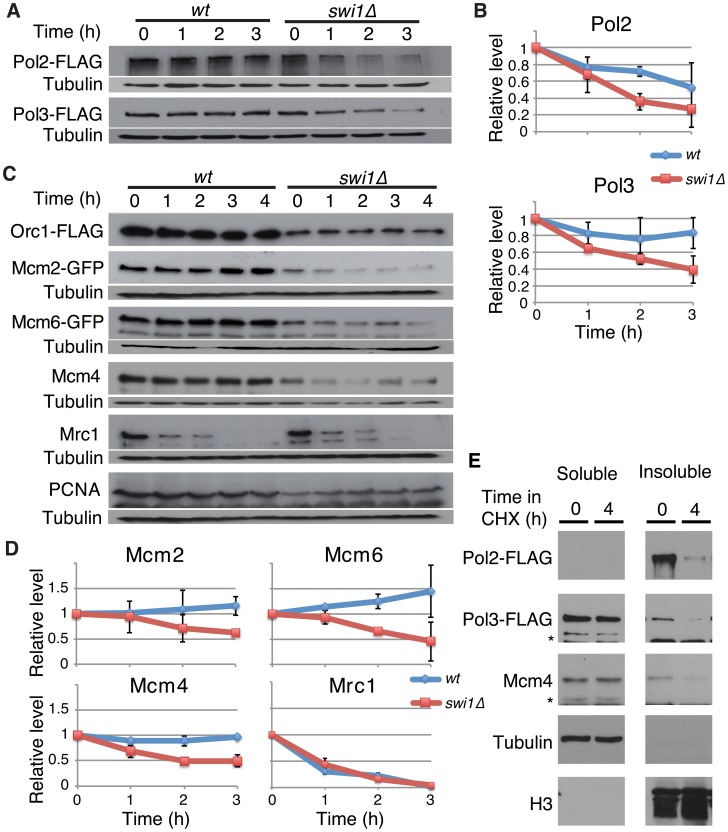
Swi1 prevents degradation of DNA polymerases and helicases. Exponentially growing cells were treated with 0.1 mg/ml CHX at 25°C. (A) Cellular amounts of Pol2-FLAG and Pol3-FLAG were examined from 0 to 3 h of CHX treatment. The anti-FLAG M2 antibody was used to detect Pol2 and Pol3. Western blotting of tubulin was also performed as a loading control. (B) Stability of Pol2-FLAG and Pol3-FLAG shown in *A* was quantified by NIH ImageJ. Relative intensity of protein bands at 0 h was set to 1 in each experiment. Error bars correspond to standard deviation of three independent experiments. *wt*, in blue; *swi1*Δ, in red. (C) Cellular amounts of Mcm2-GFP, Mcm6-GFP, Mcm4, Mrc1, Orc1-FLAG, and PCNA were determined from 0 to 4 h of CHX treatment. Anti-FLAG, anti-GFP, anti-Mcm4, anti-Mrc1, and anti-PCNA antibodies were used for Western blotting. (D) Stability of Mcm2-GFP, Mcm6-GFP, Mcm4, and Mrc1, shown in *C*, was quantified as described in *B*. Error bars represent average deviation (*n* = 2) or standard deviation (*n* = 3). (E) Replisome components in a chromatin-enriched fraction were degraded in response to CHX. Chromatin-free (Triton-soluble) and chromatin-enriched (Triton-insoluble) fractions were prepared from *S. pombe* cells treated with CHX for 0 and 4 h. The fractions were analyzed by Western blotting using antibodies to detect the indicated proteins.

Recent studies have shown that fork progression is impaired in the absence of FPC orthologues [24], [27], [28], [38], [52]. We also found a similar defect in *S. pombe swi1*Δ cells (Figure S1), suggesting that FPC might regulate replisome stability. To test this possibility, we investigated the stability of various replication proteins in cells treated with cycloheximide (CHX), a compound that blocks the synthesis of new proteins and allows for the examination of protein stability. First, we examined the stability of the catalytic subunits of major essential replicative DNA polymerases. For this purpose, we employed *S. pombe* cells expressing Pol2-FLAG (the catalytic subunit of DNA polymerase ε, required for leading-strand synthesis) [53] and Pol3-FLAG (the catalytic subunit of DNA polymerase δ, required for lagging-strand synthesis) [54] from their genomic loci. Pol2-FLAG showed significant degradation in wild-type cells, whereas, Pol3-FLAG was relatively stable (Figure 1A and 1B). Intriguingly, Pol2 displayed even faster degradation when *swi1* was deleted. In addition, Pol3 showed dramatic instability in *swi1*Δ cells (Figure 1A and 1B). Next, we examined the stability of MCM helicase components. *S. pombe* cells expressing Mcm2-GFP or Mcm6-GFP from their genomic loci were used, and Mcm4 was detected by the anti-Mcm4 antibody. In wild-type cells, Mcm2-GFP, Mcm4, and Mcm6-GFP were stable and did not undergo significant degradation throughout the CHX treatment (Figure 1C and 1D). In contrast, these helicase subunits were rapidly degraded in *swi1*Δ cells (Figure 1C and 1D). To determine whether such degradation is specific to certain replication proteins, we also assessed the stability of Orc1 (an ORC subunit), Mrc1 (a mediator of S-phase checkpoints), and PCNA. Although the steady-state levels of Orc1-FLAG and PCNA before the addition of CHX were somewhat lower in *swi1*Δ cells, their cellular amounts were maintained throughout the 4 h of CHX treatment in both wild-type and *swi1*Δ cells (Figure 1C). As previously reported [55], Mrc1 was unstable and shows rapid degradation in the presence of CHX, although this degradation was not strengthened by the deletion of *swi1* (Figure 1C and 1D). Thus, we concluded that Swi1 is involved in preventing rapid degradation of Pol2 and Pol3, as well as helicase components. Since Swi1 is involved in the suppression of fork collapse at difficult-to-replicate regions in fission yeast [43]–[45], it is possible that chromatin-bound replisome components are susceptible to degradation. Therefore, we fractionated cells into Triton-X-100-soluble (cytosol and nucleoplasm) and Triton-X-100-insoluble (enriched with chromatin- and nuclear matrix-bound proteins) fractions as described in previous studies (Figure 1E) [55], [56]. Tubulin and histone H3 were exclusively fractionated into the Triton-soluble and Triton-insoluble fractions, respectively, indicating that fractionation was successful. Pol2 was mainly fractionated into the Triton-insoluble fraction, while approximately 20% and 30% of Pol3 and Mcm4 were recovered into the Triton-insoluble fraction, respectively. Importantly, degradation of Pol2, Pol3 and Mcm4 was observed in the Triton-insoluble fraction when cells were treated with CHX, suggesting that the chromatin fraction of replisome components undergoes degradation (Figure 1E). Therefore, our results are consistent with the notion that cells promote a fast turnover of replisome components bound to chromatin in response to the accumulation of fork collapse.

### Swi1 protects the replisome components from proteasome-dependent degradation

**Figure 2:**
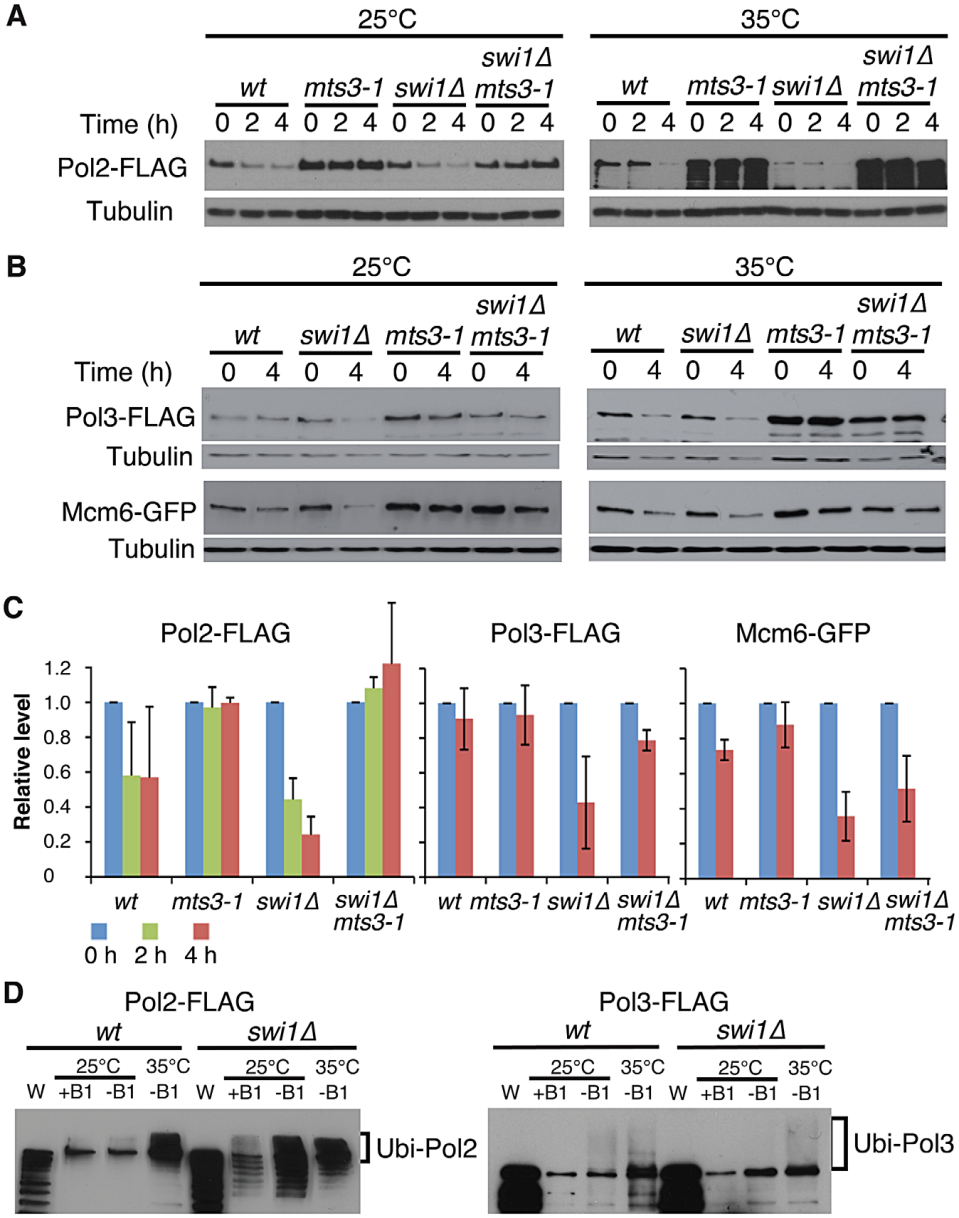
Ubiquitin-proteasome-dependent degradation of replisome core components in the absence of Swi1. (A) Inactivation of the proteasome stabilizes Pol2. Cells of the indicated genotypes were incubated for 2 h at the indicated temperatures, then treated with CHX for 4 h. Cellular levels of Pol2-FLAG were monitored after 0, 2 and 4 h of CHX treatment. Tubulin was used as a loading control. (B) Cellular levels of Pol3-FLAG and Mcm6-GFP in the indicated cells were determined after 0 and 4 h of CHX treatment as described in A. (C) Stability of Pol2-FLAG, Pol3-FLAG and Mcm6-GFP at 25°C shown in A and B was quantified. Relative intensity of protein bands at 0 h in each cell type was set to 1. Error bars correspond average deviation (*n* = 2) or standard deviation (*n* = 3). (D) Pol2 and Pol3 are highly ubiquitinated in the absence of Swi1. 6xHis-Ub peptide was expressed 22 hours in the absence of thiamine (−B1) at 25°C, then cells were placed for 2 hours at 25°C or 35°C. There is some leaking expression of 6xHis–Ub before induction in the presence of thiamin (+B1). Ubiquitinated proteins were purified as described in Materials and Methods. Western blotting of the indicated protein was performed. W, whole cell extract. Representative results of repeat experiments are shown.

To understand the mechanisms of replisome degradation in response to unstable forks in the absence of Swi1, we determined whether the proteasome is responsible for degradation of DNA helicases and polymerases. The *mts3-1* temperature-sensitive allele, which has a mutation in a subunit of the 26S proteasome machinery, was used to inactivate the proteasome [57], [58]. It is estimated that proteasome activity of *mts3-1* cells is about 50% and 30% of the wild-type enzyme at 25°C and 35°C, respectively [58]. Cells were grown at 25°C or 35°C for 2 h, and then treated with CHX for 2 to 4 h. Strikingly, degradation of Pol2 was substantially inhibited in *mts3-1* and *swi1*Δ *mts3-1* cells even at 25°C (Figure 2A and 2C). We observed similar stabilization of Pol3 and Mcm6 in *mts3-1* and *swi1*Δ *mts3-1* cells (Figure 2B and 2C). At 35°C, degradation of these replisome components was accelerated both in wild type and *swi1*Δ cells probably due to increased cell metabolism (Figure 2A and 2B). However, degradation of these replisome components was abolished in *mts3-1* and *swi1*Δ *mts3-1* cells at 35°C (Figure 2A and 2B). Thus, our data indicate that Swi1 prevents proteasome-dependent degradation of replisome components.

Ubiquitin moieties (Ub) are conjugated to most of the proteins degraded by the proteasome [59], . Therefore, aforementioned data suggest that replisome core components (polymerases and helicases) are ubiquitinated. To test this possibility and further understand the mechanism of replisome degradation, we investigated whether replisome components were ubiquitinated. Cells harboring FLAG-tagged versions of Pol2 and Pol3 were engineered to express hexahistidine-fused ubiquitin (6xHis–Ub peptide) under the control of the thiamine (B1)-repressible *nmt1* promoter. They were first cultured in the presence of thiamin (B1) to repress the *nmt1* promoter and then grown in the absence of thiamine for 22 h at 25°C, allowing cells to express 6xHis–Ub peptide. After the 22 h incubation, cultures were divided and further incubated at 25°C or 35°C for 2 h. Ubiquitinated proteins were purified with nickel agarose beads and analyzed by immunoblotting using antibodies against the FLAG-tag (Figure 2B). As shown in Figure 2D, Pol2-FLAG species with slower gel mobility were clearly detected in both wild type and *swi1*Δ cells, indicating that Pol2 is ubiquitinated. We also observed precipitation of non-ubiquitinated Pol2 with nickel agarose as previously reported for other proteins [61]. In addition, multiple Pol2 bands, which are probably products of degraded Pol2, were detected in *swi1*Δ cells (Figure 2D), suggesting that Pol2 is more susceptible to degradation in the absence of Swi1. Similarly, ubiquitinated forms of Pol3-FLAG were detected in wild type and *swi1*Δ cells (Figure 2D). However, with our methods, we were not able to observe ubiquitinated forms of Mcm proteins (data not shown). Considering that Mcm proteins are stabilized in *mts3-1* cells (Figure 2A), it is possible that the ubiquitination and degradation processes of Mcm proteins are too rapid to be detected.

### Pol2 degradation occurs during S phase and is dependent on SCF^Pof3^

**Figure 3:**
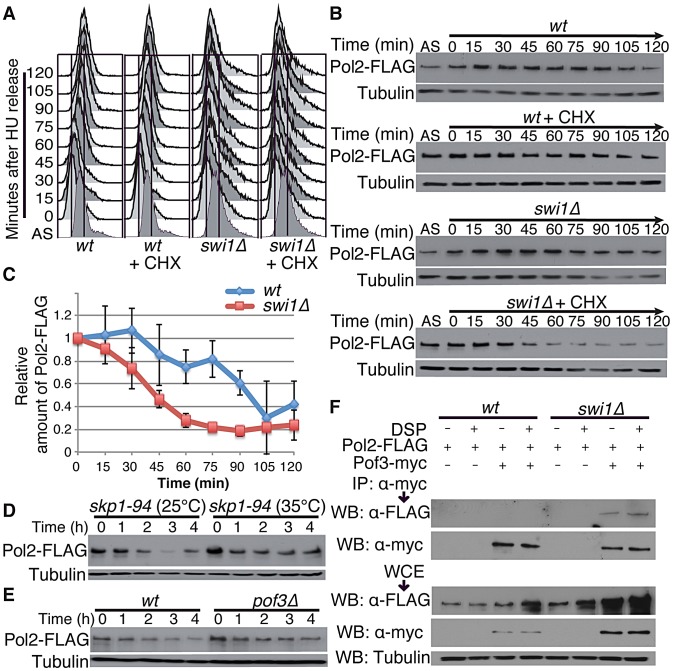
Pol2 degradation occurs in S phase and is SCF^Pof3^ dependent. Exponentially growing cells (AS) were synchronized at the G_1_/S (time zero) boundary in the presence of 12 mM HU for 3 h, and were released into fresh YES medium with or without CHX. Cells were collected and processed for DNA content analysis in *A*, and for Western blotting in *B*. (A) Cells were fixed at the indicated times, and DNA contents were analyzed by flow cytometry. (B) Pol2 is degraded during S phase in *swi1* mutants. Cellular amounts of Pol2-FLAG were determined at the indicated times. Tubulin levels were also monitored as a loading control. Representative results of repeat experiments are shown. (C) Stability of Pol2-FLAG during the CHX treatment shown in *B* was quantified as described in Figure 1. For each strain, relative intensity of the Pol2-FLAG band at 0 h was set to 1. (D) Pol2 is stabilized in the *skp1-94* mutants. Exponentially growing *skp1-94* cells were treated with CHX at 25°C and 35°C. Cellular amounts of Pol2-FLAG were examined from 0 to 4 h of CHX treatment. (E) *pof3* deletion stabilizes Pol2. As in *D*, Pol2-FLAG levels were examined during the CHX treatment of wild-type and *pof3*Δ cells. (F) Pof3 interacts with Pol2. Cells expressing the indicated fusion proteins (Pol2-FLAG and/or Pof3-Myc) were harvested in the presence or absence of DSP, and protein extracts were prepared. Pof3-Myc was immunoprecipitated, and associated proteins were probed with anti-FLAG antibody. Representative results of repeat experiments are shown. IP, immunoprecipitation; WB, Western blotting; WCE, whole cell extract.

Swi1 and its orthologues are involved in DNA replication, and their defects cause replication stress at difficult-to-replicate genome regions [14], [15], [18], [21], [22], [24], [25], [27], [28], [43]–[45]. Thus, our results suggest that replisome core degradation occurs during S phase in the absence of Swi1. To test this possibility, wild type and *swi1*Δ cells were synchronized at the G_1_/S boundary in the presence of 12 mM hydroxyurea (HU) and released into S phase in fresh medium supplemented with CHX. FACS analysis showed that the addition of CHX did not perturb cell cycle progression through S phase after the removal of HU (Figure 3A). There was no significant Pol2 degradation in both wild type and *swi1*Δ cells in the absence of CHX. In contrast, the level of Pol2-FLAG dramatically dropped between 30 and 45 min after CHX addition in the absence of Swi1 (Figure 3B and 3C), when cells are in S phase (Figure 3A). In contrast, wild-type cells displayed only a mild decrease in the level of Pol2-FLAG (Figure 3B and 3C). We also used the *cdc25-22* temperature sensitive allele to synchronize cells at the G_2_/M boundary at the restrictive temperature (35°C), and cells were released into the cell cycle at permissive temperature (25°C). As determined by the increase of septation index, cells synchronously entered S phase after the release in the absence of CHX (Figure S2A). In this condition, Pol2 levels were maintained throughout the experiments in both *cdc25-22* and *cdc25-22 swi1*Δ cells (Figure S2B and S2C). When cells were treated with CHX, Pol2-FLAG levels gradually decreased in *cdc25-22 swi1*Δ cells but not in *cdc25-22* cells (Figure S2B and S2C). This mild degradation is probably because cells were unable to synchronously progress through S phase in the presence of CHX (Figure S2A), although our data indicate that Pol2-FLAG is unstable in *swi1*Δ cells. Interestingly, Mcm4 showed rapid degradation as *cdc25-22 swi1*Δ cells progress through S phase in the absence of CHX (Figure S2B and S2C), indicating that Mcm4 is degraded during replication. Mcm4 degradation in *cdc25-22 swi1*Δ cells was further exacerbated in the presence of CHX. In contrast, there is no significant Mcm4 degradation in *cdc25-22* cells with or without CHX treatment (Figure S2B and S2C). Taken together, we concluded that degradation of replisome core components occurs during DNA replication in the absence of Swi1.

Since SCF ubiquitin ligases are often involved in protein degradation during S phase [62], we examined the stability of Pol2 in *skp1-94* temperature-sensitive cells, which have a mutation in Skp1, a major component of SCF ubiquitin ligases in *S. pombe* [63]. Strikingly, Pol2-FLAG was significantly stabilized when *skp1-94* cells were incubated at 35°C, indicating the involvement of SCF ubiquitin ligases in Pol2 degradation (Figure 3D; Figure S3B). SCF ligases contain F-box subunits, which are responsible for substrate specificity. Therefore, we examined Pol2 stability in a series of mutants defective for F-box proteins (Figure S3). Among the eleven F-box mutants we tested, we found that Pol2 becomes most stable in the absence of Pof3 (Figure 3E; Figure S3), an F-box protein that has been suggested to be involved in the preservation of genomic integrity [64]. Thus, our data suggest that Pol2 degradation is in part mediated by the SCF^Pof3^ ubiquitin ligase.

To further understand the mechanism of Pol2 degradation, we examined whether Pof3 interacts with Pol2, using immunoprecipitation assays. Cells expressing Pol2-FLAG proteins were engineered to express Pof3-Myc from its genomic locus. As shown in Figure 3F, Pol2-FLAG coprecipitated with Pof3-Myc in the absence of Swi1, indicating that SCF^Pof3^ interacts with Pol2. The Pol2–Pof3 interaction was not detectable in wild-type cells even in the presence of a protein crosslinker dithio-bis succinimidyl propionate (DSP) (Figure 3F). Therefore, our data suggest that SCF^Pof3^–Pol2 interaction is transient in wild-type cells but is enhanced when Pol2 degradation is accelerated in the absence of Swi1.

### SCF^Pof3^ is involved in degradation of Mcm4 and Mrc1

**Figure 4:**
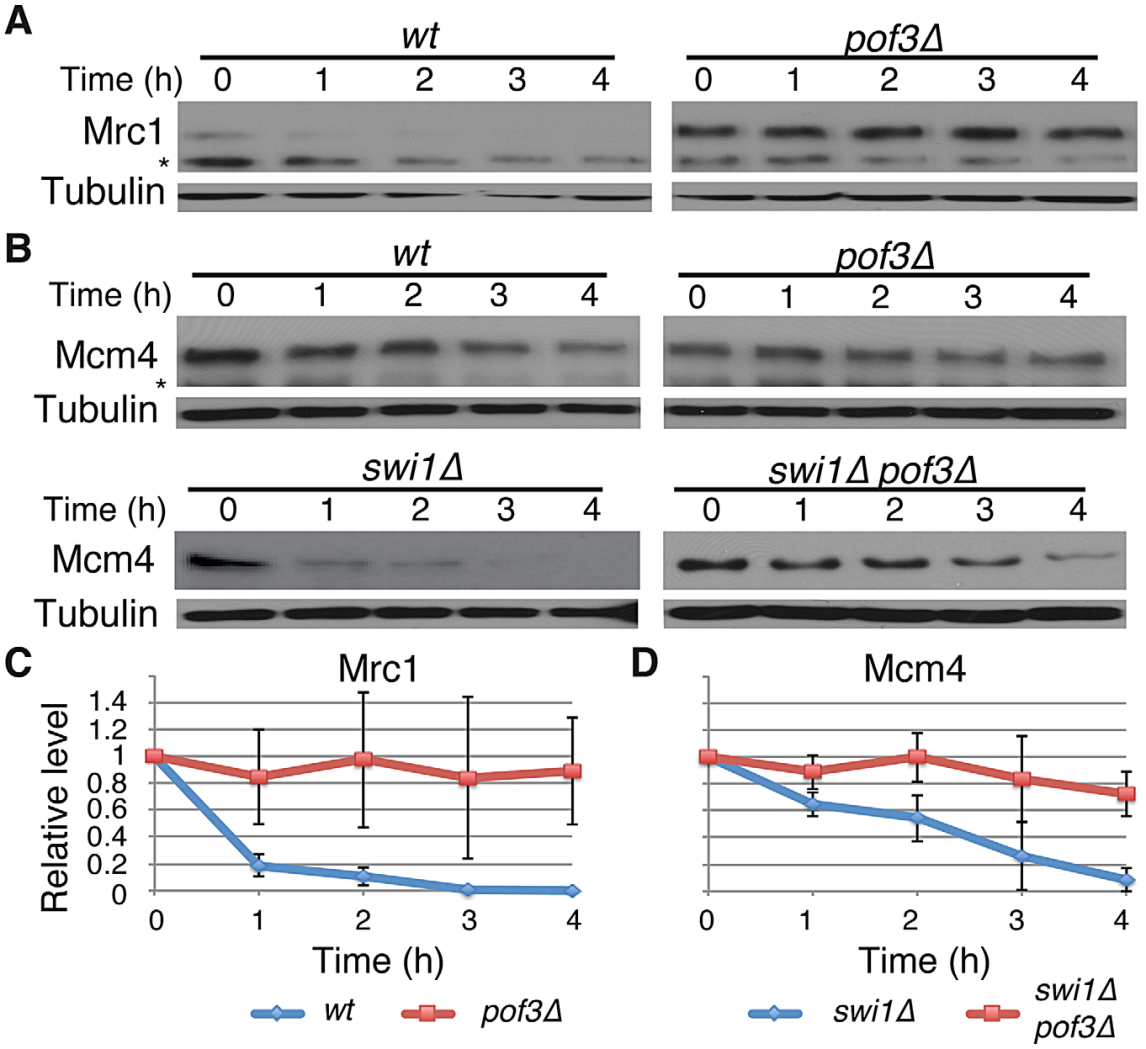
Pof3-dependent degradation of Mrc1 and Mcm4. (A) Cellular amount of Mrc1 was determined in *wt* and *pof3*Δ cells, from 0 to 4 h of CHX treatment. Western blotting of tubulin was performed as a loading control. (B) As in *A*, cellular amount of Mcm4 was determined in *wt*, *pof3*Δ, *swi1*Δ, and *pof3*Δ *swi1*Δ cells. The asterisks indicate non-specific bands. (C) Stability of Mrc1 during the CHX treatment shown in *A* was quantified as described in Figure 1. For each strain, relative intensity of the Mrc1 band at 0 h was set to 1. (D) Stability of Mcm4 in *swi1*Δ and *pof3*Δ *swi1*Δ shown in *B* was quantified as described in *C*. Samples for Mrc1 or Mcm4 blots were derived from the same experiment and processed in parallel. Representative results of repeat experiments are shown.

SCF^Pof3^ has been shown to interact with fission yeast Mcl1, a DNA polymerase α accessory factor related to budding yeast Ctf4 [64]–. Moreover, in budding yeast, Dia2 (Pof3-related protein) is recruited to the replication fork [67], [68] and is involved in the ubiquitination of Mrc1 [69]. Therefore, SCF^Pof3^-dependent Pol2 degradation suggests that SCF^Pof3^ may also target other replisome components for degradation. We first sought to determine whether Pof3 is also involved in degradation of Mrc1 in *S. pombe*. As shown in Figure 4, Mrc1 became highly stable in *pof3*Δ cells under CHX treatment (Figure 4A and 4C). We then examined whether Mcm4 degradation in *swi1*Δ cells is inhibited by the inactivation of SCF^Pof3^ (Figure 4B and 4D). Intriguingly, Mcm4 was significantly more stable in *pof3*Δ *swi1*Δ cells than in *swi1*Δ cells after CHX treatment. This result suggests that SCF^Pof3^ also targets Mcm4 for proteasome-dependent degradation in response to replication stress provoked by *swi1* deletion. Taken together, our results are consistent with the notion that SCF^Pof3^ is involved in degradation of multiple replisome components.

### Replisome degradation prevents mitotic catastrophes in *swi1*Δ cells

**Figure 5:**
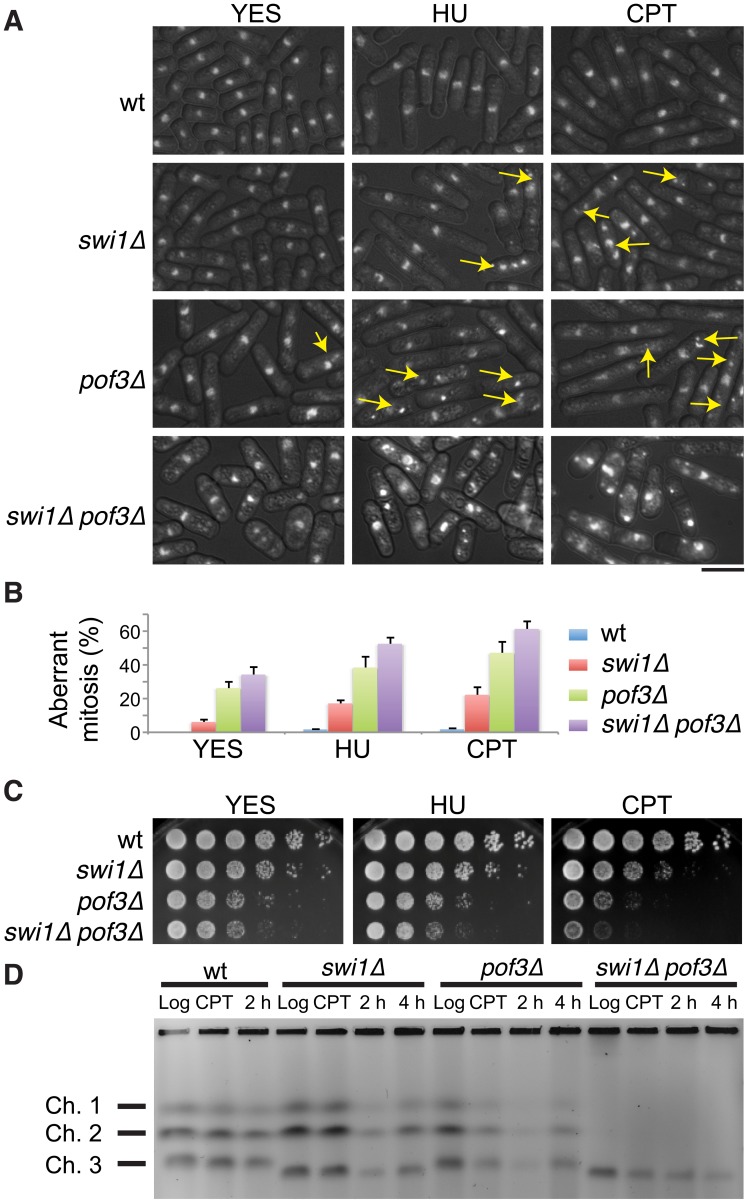
Forced accumulation of replisome components in *swi1*Δ cells causes catastrophic DNA replication and mitotic abnormalities. (A) *swi1*Δ *pof3*Δ cells have increased levels of mitotic catastrophes. Exponentially growing cells were treated with or without the indicated drugs (12 mM HU or 20 µM CPT for 6 h), fixed in ethanol, and stained with 4′,6-diamidino-2-phenylindole (DAPI). Representative images of observed nuclear phenotypes are shown. Representative mitotic failures are shown by arrows. Arrows were omitted from the images of *swi1*Δ *pof3*Δ cells because a large numbers of cells showed mitotic catastrophes. The scale bar represents 10 µM. (B) Quantification of cells with defective mitosis including chromosome missegregation, aneuploidy, cut and other aberrant phenotypes. More than 200 cells were counted for each strain. Error bars correspond to standard deviations obtained from three experiments. (C) DNA damage sensitivity of *swi1*Δ mutants is increased by *pof3* deletion. Five-fold serial dilutions of cells were incubated on YES agar medium supplemented with the indicated drugs (2 mM HU or 1 µM CPT) for 2 to 3 days at 32°C. (D) *pof3* deletion exacerbates replication recovery defects of *swi1*Δ mutants. Exponentially growing cells (Log) were incubated in the presence of 5 µM CPT for 3 h at 30°C (CPT), then washed and returned into fresh medium for 2 h or 4 h (2 h, 4 h). Chromosome samples were examined by PFGE. Representative results of repeat experiments are shown. *swi1*Δ cells have shorter chromosome III due to hyper recombination at rDNA repeats [42], [70], [101].

In order to understand the physiological importance of replisome core degradation in the absence of Swi1, we investigated cellular phenotypes of *swi1*Δ, *pof3*Δ and *swi1*Δ *pof3*Δ double mutant cells. For this purpose, cells were stained with DAPI to visualize nuclear DNA. As shown in Figure 5A and 5B, *swi1*Δ and *pof3*Δ cells displayed an increased level of mitotic catastrophes (including chromosome missegregation, aneuploidy, fragmented nuclei, hypercondensed nuclei, “cut” and other aberrant phenotypes, which are shown by arrows) compared to wild-type cells. Importantly, this phenotype was further exacerbated in *swi1*Δ *pof3*Δ cells even in the absence of exogenous genotoxic agents (Figure 5A and 5B). We then used HU and camptothecin (CPT) to introduce S phase specific genotoxic stress. HU depletes the dNTP pool and causes an arrest of replication fork progression, while CPT traps topoisomerase I on DNA and induces replication fork breakage. HU or CPT treatment further enhanced the aberrant mitotic phenotypes (Figure 5A and 5B). Consistently, *swi1*Δ *pof3*Δ cells were more sensitive to HU and CPT than either single mutant (Figure 5C). In the presence of HU or CPT, *swi1*Δ cells accumulate DNA damage due to failure in the completion of DNA replication, which causes activation of the DNA damage checkpoint, leading to abnormal cell cycle arrest and a cell elongation phenotype [17], [70]. As expected, HU or CPT treatment caused cell elongation in *swi1*Δ cells (Figure 5A). However, this elongation phenotype was abolished in *swi1*Δ *pof3*Δ cells (Figure 5A), suggesting that the stabilization of replisome components attenuated cell cycle arrest in *swi1*Δ *pof3*Δ cells. This attenuation of cell cycle arrest could have caused a growth advantage, leading to the rather weak increase in the HU and CPT sensitivity of *swi1*Δ *pof3*Δ cells (Figure 5C), although these cells show strong mitotic catastrophes (Figure 5A).

**Figure 6:**
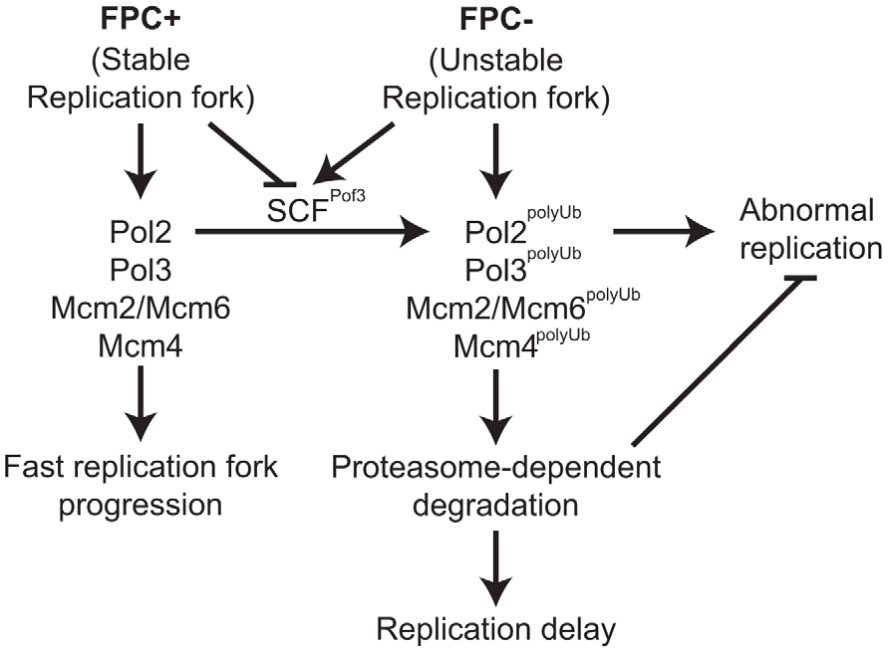
Degradation of replisome components prevents genomic instability. Models for the roles of the FPC in replisome stabilization. The FPC (Swi1-Swi3) stabilizes the replication fork (*left*; FPC+) and replisome components and promotes an efficient progression of the replication fork. The FPC suppresses replication stress that leads to unstable replication forks (*right*; FPC−). Under replication stress, SCF^Pof3^ may have access to replisome components and transfer ubiquitin moieties. Ubiquitinated replisome components undergo proteasome-dependent degradation, resulting in delayed replication fork progression. Replisome degradation prevents abnormal DNA replication, thus preserving genomic integrity.

Next, we examined the ability of cells to recover DNA replication after CPT-dependent replication fork breakage. Exponentially growing cells (Log) were exposed to a low dose of CPT (5 µM) for 3 h and returned to fresh medium for 2 and 4 h (Figure 5D). Chromosome samples were then analyzed by pulsed-field gel electrophoresis (PFGE), which permits only fully replicated chromosomes to migrate into the gel. In contrast, chromosomes with replication intermediates stay in the well of the gel, allowing us to determine the rate of replication recovery. There was no detectable DNA replication defect in wild-type cells throughout the experiment (Figure 5D, Log, CPT, 2 h), indicating that the low dose of CPT used in this assay did not cause major replication problems in wild-type cells. Although chromosomes from *swi1*Δ cells migrated into the gel immediately after the CPT exposure (CPT), we observed a marked reduction in chromosome intensity at 2 h after CPT treatment (Figure 5D). This result indicates that the low dose of CPT caused replication problems in *swi1*Δ cells, which is consistent with previous studies [70]. However, there was a significant recovery at 4 h after the CPT removal due to the completion of DNA synthesis. A similar replication recovery was also observed in *pof3*Δ cells. In contrast, there was no DNA replication recovery in *swi1*Δ *pof3*Δ cells during the course of the experiment (Figure 5D), indicating that these cells experience further difficulties in replication and/or repair of broken replication forks when treated with a replication-stressing agent. Interestingly, we repeatedly observed much less appearance of chromosomes I and II in the gel for *swi1*Δ *pof3*Δ cells (Figure 5D; Figure S4), suggesting that these cells experience major problems in DNA replication and chromosome maintenance. Consistently, there was an increased level of mitotic catastrophes in these cells (Figure 5A and 5B). Considering that *pof3* deletion stabilizes replisome components (Figure 3E; Figure 4), our results suggest that programmed replisome degradation represents a mechanism to prevent catastrophic DNA replication in response to replication stress caused by *swi1* deletion (Figure 6). Similar replication and mitotic phenotypes were observed in *swi1*Δ *mts3-1* cells, which are defective in proteasome functions (Figure S5), strengthening the idea that replisome degradation plays a critical role in the maintenance of genomic integrity.

## Discussion

Accurate transmission of genetic information is one of the major tasks cells need to achieve in order to preserve the species and prevent genetic diseases. Accordingly, eukaryotic cells have developed a variety of genome maintenance mechanisms. In response to DNA damage or replication stress, cells activate checkpoint pathways to coordinate cell cycle arrest with DNA repair activities. It is also known that the replication checkpoint functions to stabilize replication forks by preserving replisome and DNA structures. In this study, we have described an alternative cellular mechanism in response to replication stress. Our studies suggest that cells facilitate proteasome-dependent degradation of replisome components in response to replication stress to preserve genomic integrity.

### Proteasome-dependent degradation of replisome components preserves genomic integrity

Swi1 and its orthologues are known to be involved in the stabilization of replication forks to prevent genetic instability during DNA replication. Genetic analyses have suggested that FPC is involved in coordinating leading- and lagging-strand DNA synthesis. It is also suggested that the FPC couples polymerase and helicase activities at stalled forks [14], [15]. Thus, the functions of Swi1 would become even more important to maintain the integrity of the replication fork when it encounters difficult-to-replicate sites or programmed fork pausing sites that are scattered throughout the genome. Consistently, FPC plays a critical role in programmed fork pausing and replication termination events near the mating-type (*mat1*) locus and at fork pausing sites in rDNA repeats and tRNA loci in yeast [16], [17], [21], [29], [38], [39]. Importantly, recent studies indicated that *swi1*Δ cells experience fork collapse at these difficult-to-replicate regions [43]–[45]. Therefore, inactivation of Swi1 causes defects in replication fork stabilization at natural impediments, leading to general replication stress at the replication fork.

It is well known that Cdt1 and Cdc6 undergo rapid proteasome-dependent degradation to restrict replication licensing once per cell cycle [71], [72]. However, how replisome degradation contributes to DNA replication process is largely unknown. In this report, we show that DNA polymerases and helicases undergo rapid degradation upon replication stress in the absence of Swi1 (Figure 1). This degradation is dependent on the ubiquitin-proteasome system (Figure 2). In the absence of Swi1, cells experience unstable replication forks that lead to an increased level of replication-dependent DNA damage and hyper-recombination [16], [17], [43]. Such replication stress appears to cause replisome degradation in order to prevent abnormal DNA replication and mitotic catastrophes (Figure 5; Figure S5). These results suggest that replisome degradation functions to maintain genomic integrity during DNA replication in response to replication stress (Figure 6). Similar mechanisms have been described in the transcription-coupled DNA repair (TCR), which is activated by transcription blockage in response to genotoxic agents [73], [74]. In this mechanism, the Cockayne syndrome B protein (budding yeast Rad26) interacts with Def1 to regulate ubiquitination of Rpb1, the large subunit of RNA polymerase II (RNAPII), which results in proteasome-dependent degradation of RNAPII [75],[76]. Ubiquitination of Rbp1 is achieved by the Rsp5/Nedd4 ubiquitin ligase, which promotes DNA-damage induced degradation of RNAPII in budding yeast and human cells [77]–[79]. RNAPII degradation appears to be an alternative mechanism to TCR. Studies indicate that the loss of both TCR and RNAPII degradation pathways renders cells hypersensitive to DNA damage, thus Def1 promotes proteolysis of RNAPII when the lesion cannot be rapidly repaired by TCR [75], [80]–[82]. Therefore, analogous to the DNA damage-induced RNAPII degradation pathway, our present findings suggest that the cell elicits a replisome degradation program when the replication fork is adversely blocked. We speculate that, depending on the degree of replication problems, re-building and re-loading new replisomes might be advantageous to the cell, rather than re-using existing replisome components that are compromised. Therefore, we suggest that replisome degradation is an alternative mechanism to replisome stabilization and prevents DNA synthesis by compromised replisomes.

### Roles of Pol2 degradation in replisome dynamics

We also found that Pol2 (Polε) is significantly unstable even in wild-type cells (Figure 1A and 1B), while Pol3 (Polδ) is relatively stable (Figure 1A and 1B). The high rate of Pol2 turnover may suggest that Pol2 needs to be re-loaded during leading-strand synthesis. Since Pol2 is suggested to work continuously on the leading-strand [53], one might think that the high turnover of Pol2 poses a disadvantage to the cells. However, it is possible that the polymerases fall off the chromatin every time the fork arrives at programmed pausing sites or difficult-to-replicate regions. In addition, Pol2 may undergo degradation once it falls off the chromatin. Such a degradation mechanism would also be advantageous for the cell to refresh Pol2 enzymes by efficiently reloading newly synthesized Pol2 at the moving replication fork. On the other hand, the discontinuous nature of Pol3-dependent lagging-strand synthesis would be sufficient to keep Pol3 refreshed at the fork in order to avoid replication-dependent errors or mutations. Another possibility is that this mechanism might simply maintain the coupling of leading- and lagging-strand synthesis. Thus, in addition to the role of replisome degradation in preventing genomic instability described above, polymerase degradation may function to eliminate non-functional replisomes and serve as a mechanism to maintain active DNA polymerases at the replication fork.

Our investigation also revealed that Pol2 and Mcm4 undergo rapid degradation in the presence of CPT (Figure S6), which breaks replication forks. However, in this condition, the Mcm6 level was maintained (Figure S6), although it was highly unstable in *swi1*Δ cells (Figure 1C and D). It is possible that some replisome components remain stable on the chromatin in the presence of CPT. Interestingly, Trenz et al. reported that polymerases fall off the chromatin in response to CPT, whereas Mcm7 persists [83]. Therefore, *swi1* deletion generates a situation distinct from a simple mechanical breakage of the fork caused by DNA damaging agents, where the replisome cannot continue replicating DNA. Importantly, Swi1 functions as an ancillary component of the replisome by interacting with various replisome components, coupling polymerase and helicase activities and coordinating semi-discontinuous DNA synthesis [14], [15]. It is also reported that Swi1 protects replication forks at difficult-to-replicate sites [43]–[45]. Therefore, we suggest that the loss of Swi1 results in unstable replisome structures at the moving replication fork during ongoing DNA synthesis, allowing us to examine replisome degradation pathways during DNA replication.

### The FPC–dependent stabilization of replisome components

The FPC moves with the replication fork and interacts with replisome components [16], [18], [21]–[25], [27], [28], [84]–[87]. Surprisingly, Pol2, Pol3, and MCM subunits are rapidly degraded in *swi1*Δ cells (Figure 1). Consistently, replication fork progression is compromised in FPC deficient cells (Figure S1) [27], [52]. These results suggest that Swi1 prevents degradation of replisome components to maintain efficient progression of replication forks. In wild-type cells, multiple activities required for DNA synthesis are coupled to form a large replisome complex, resulting in efficient progression of the replication fork. However, in the absence of Swi1, DNA replication-related activities are probably uncoupled especially at naturally difficult-to-replicate regions. This uncoupling generates unstable replisome structures, which may expose degradation signals of various replisome components to a ubiquitin ligase(s) associated with the replication fork. Importantly, *swi1*Δ *pof3*Δ double mutants showed catastrophic DNA replication and mitosis, suggesting that Pof3-dependent degradation of replisome components prevents genomic instability. However, we cannot exclude the possibility that mitotic catastrophe phenotypes are caused by stabilization of other Pof3 targets. For example, Pof3-dependent proteolysis of Ams2 is responsible for cell cycle-dependent transcriptional activation of core histone genes in *S. pombe* [88]. Indeed, defects in Ams2 degradation leads to accumulation of histones and alteration of centromere structures [88]. Such dysregulation of histone homeostasis during S phase could also lead to abnormal DNA replication, leading to mitotic problems. However, Dia2, a Pof3-related F-box protein, is associated with the replisome and regulates replication forks in budding yeast. Dia2 is involved in ubiquitination of budding yeast Mrc1, which is a component of the replisome [67]–[69]. Moreover, Tof1 (Swi1 orthologue) collaborates with Dia2 to maintain genomic integrity [89]. These findings suggest that Pof3/Dia2 acts as a part of the replisome. Consistently, we found in fission yeast that SCF^Pof3^ is largely responsible for degradation of some replisome components (Figure 3 and Figure 4). Therefore, Pof3-mediated ubiquitination of replisome components may be prevented by Swi1-dependent replisome stabilization, which may mask potential degradation signals of multiple replisome components (Figure 6). Since many SCF ubiquitin ligases are known to recognize phosphorylated degradation signals (phospho-degrons), it is also possible that replisome components undergo phosphorylation in the absence of Swi1. Therefore, Swi1 might have direct functions in inhibiting SCF^Pof3^ ligase possibly by inhibiting Pof3 or inhibiting potential kinases. In this regard, it is interesting to note that Mrc1 contains a potential phospho-degron, and that the Hsk1 kinase is required for efficient degradation of Mrc1 [55]. Consistently, our present study shows that Pof3 is involved in Mrc1 degradation (Figure 4). Therefore, it is possible that Hsk1-dependent phosphorylation creates Pof3-targeted phospho-degrons on multiple replisome components. However, Mrc1 degradation is independent of replication stress (Figure 1C), raising the possibility that other kinases are responsible for replisome degradation upon replication stress. Further investigation of proteasome-dependent replisome degradation would identify detailed pathways in the regulation of the replisome.

## Materials and Methods

### General techniques

The methods used for genetic and biochemical analyses of fission yeast have been described previously [90], [91]. Drug sensitivity assays, Western blotting, pulsed-field gel electrophoresis (PFGE) and 4′,6-diamidino-2-phenylindole (DAPI) staining of nuclear DNA were performed as described [70], [92]. Flow cytometry of DNA content has been described [93], [94].

### *S. pombe* strains

*S. pombe* strains used in this study were constructed using standard techniques [91], and their genotypes and sources are listed in Table S1. *swi1*Δ (*swi1*::*hphMX6* and *swi1*::*natMX6*) and *pof3*Δ (*pof3*::*ura4MX6*) were generated by a two-step PCR method [95], to replace *swi1* and *pof3* open reading frames with selection marker genes. The two-step PCR method was also used to construct a GFP or 13Myc tag at the C terminus of *mcm2*, *mcm6* and *pof3*, generating *mcm2-GFP*:*hphMX6* (*mcm2-GFP*), *mcm6-GFP*:*hphMX6* (*mcm6-GFP*), and *pof3-13Myc*:*hphMX6* (*pof3-13Myc*), respectively. Oligonucleotide primers used in the two-step PCR method described above are listed in Table S2. A temperature-sensitive *skp1-94* mutation was isolated using error-prone PCR methods [63].

Mutations and epitope-tagged genes have been described for *orc1-5FLAG* [96], *pol2-5FLAG*, *pol3-5FLAG* [97], *swi1*::*kanMX6* [17], *cdc45-5FLAG* [98], *cdc25-22* [99] and *mts3-1* [57].

*mcm2-GFP*, *mcm6-GFP*, *pof3-13MYC*, *orc1-5FLAG*, *pol2-5FLAG*, *pol3-5FLAG*, and *cdc45-5FLAG* cells show normal growth phenotype and were not abnormally sensitive to HU, CPT and MMS, indicating that the tagged version of these proteins are functional.

### Cell extract preparation for Western blotting

To examine protein stability, exponentially growing cells were treated with 0.1 mg/ml of cycloheximide (CHX) for the indicated times and collected. Whole-cell extracts were prepared as described [100]. Briefly, cells were washed in STOP buffer (150 mM NaCl, 50 mM NaF, 10 mM EDTA, and 1 mM NaN_3_) and lysed by glass beads in lysis buffer U (50 mM Tris-HCl pH 6.8, 2% SDS, 2 mM EDTA, 10% glycerol, and 4 M urea) using a FastPrep Cell disruptor (Qbiogene, Irvine, CA) for 40 seconds at speed 6. Protein extract was clarified by centrifugation at 13,000 rpm in an Eppendorf microcentrifuge 5415R for 10 min at 4°C, and the protein concentration was determined using BCA protein Assay Reagent (Thermo Fisher Scientific, Waltham, MA). Immediately after the protein concentration assay, protein extracts were boiled in the presence of 5% beta-mercaptoethanol and stored at −20°C. For immunoblotting, Myc, GFP, and FLAG fusion proteins were probed with the anti-c-Myc 9E10 antibody (Covance, Princeton, NJ), anti-GFP antibody (Roche, Indianapolis, IN), and anti-FLAG M2 (Sigma-Aldrich) antibody, respectively. The anti-tubulin TAT-1 (gift from Dr. K. Gull), anti-Mcm4 (gift from Drs. S. Kearsey, Z. Lygerou, and H. Nishitani), anti-Mrc1 (gift from Dr. K. Tanaka), and anti-PCNA (gift from Dr. T. Tsurimoto) antibodies were used to detect the corresponding proteins. Quantification of protein bands was performed using NIH ImageJ software.

### Fractionation of cells into soluble and chromatin-enriched fractions

Cell fractionation was performed as described elsewhere [55], [56] with modifications. Exponentially growing cells were harvested in 0.01% sodium azide by centrifugation and washed sequentially with STOP buffer, water, and 1.2 M sorbitol, at 4°C. Cells were resuspended in CB1 buffer (50 mM sodium citrate, 40 mM EDTA, 1.2 M sorbitol) and treated with 2.5 mg/ml of Zymolyase for approximately 20 min at 32°C. When cell lysis reached approximately 95%, cell wall digestion by Zymolyase was terminated by adding equal volume of ice-cold CB2 buffer (1.2 M sorbitol, 10 mM Tris-HCl ph7.5), and resulting spheroplasts were washed twice with 1.2 M Sorbitol. Spheroplasts were then incubated in Lysis buffer T (50 mM potassium acetate, 2 mM MgCl2, 20 mM HEPES-KOH pH 7.4, 10 mM EDTA, 1 M Sorbitol, 1% Triton X-100) supplemented with Halt protease inhibitor cocktail (Thermo Fisher Scientific) for 10 min at 4°C. Subsequently, extracts were fractionated into soluble and pellet fractions by centrifugation for 10 min at 4°C. Supernatants (Triton X-100-soluble fraction) were removed, boiled with a one-third volume of 3× SDS-PAGE loading buffer (150 mM Tris-HCl pH 6.8, 6% SDS, 6 mM EDTA, 30% glycerol, 15% beta-mercaptoethanol), and stored at −20°C. The pellets (Triton X-100-insoluble fraction) were washed once with Lysis buffer (without Triton X-100), suspended in Lysis buffer, boiled with a one-third volume of 3× SDS-PAGE loading buffer, and stored at −20°C.

### Immunoprecipitation and detection of ubiquitinated proteins

Immunoprecipitation was performed using the anti-myc 9E10 (Covance) antibody with protein G sepharose beads as described [70]. Proteins associated with the anti-myc antibody were analyzed by Western blotting. For detection of ubiquitinated protein, *S. pombe* cells expressing a hexahistidine-ubiquitin (6xHis-Ub) peptide [61] were lysed in lysis buffer G (6 M guanidine hydrochloride, 100 mM sodium phosphate pH 8.0, and 50 mM Tris-HCl pH 8.0). Hexahistidine-ubiquitinated proteins were purified with Ni-NTA agarose beads (Qiagen, Valencia, CA), eluted in the presence of 4 M urea, and analyzed by Western blotting.

## Supporting Information

Figure S1 Replisome progression is delayed in the absence of Swi1. (A) Diagram of the replication origin 2004 (*ori2004*) region used in chromatin immunoprecipitation. Protein association was monitored at *ori2004* and a position 30 kb away from *ori2004* as described [16], [97]. (B) Septation index. *cdc25-22* cells were synchronized at the G_2_/M boundary at 35°C for 3 hours and released at 25°C. The septation index was determined to monitor cell cycle progression. *swi1*Δ (green) cells have a 20 min delay in the increase of septation index when compared to wild-type (*swi1^+^*; red) cells. In *S. pombe*, an increase in septation index coincides with the onset of S-phase [91]. In order to remove this difference in our analysis, we set the point of septation increase (40 min in *swi1* ^+^ and 60 min in *swi1*Δ) to 0 (S) min as the onset of S-phase in *C* and *D*. (C,D) Relative enrichment of replication proteins at the *ori2004* region during 120 min from the onset of S-phase (0 (S) min). (C) Association of Cdc45-FLAG with chromatin was monitored at *ori2004* (blue) and at a position 30 kb (green) away from *ori2004*, to evaluate their translocation through the *ori2004* region in wild-type (top panel) and *swi1*Δ cells (bottom panel). (D) As in *C*, chromatin recruitment of Pol2-FLAG was monitored through the *ori2004* region in wild-type (top panel) and *swi1*Δ cells (bottom panel). Representative results of repeat experiments are shown.
(EPS)

Figure S2 Stability of Pol2 and Mcm4 during the cell cycle. *cdc25-22* cells were synchronized at the G_2_/M boundary by incubation at 35°C for 3 h and then released into fresh YES medium with or without CHX at 25°C. (A) An increase in the septation index indicates the onset of S-phase. Cells entered S phase synchronously in the absence of CHX. However CHX affected cell cycle progression after the release from G_2_/M. (B) Cellular amounts of Pol2-FLAG and Mcm4 were determined at the indicated times after the release from G_2_/M. (C) Stability of Pol2-FLAG and Mcm4 shown in B was quantified as described in Figure 1. Pol2-FLAG was unstable in *swi1*Δ cells in response to CHX. Mcm4 showed rapid degradation during S-phase with or without CHX.
(EPS)

Figure S3 Quantification of Pol2-FLAG stability in F-box mutants. (A) Pol2 degradation was examined in wild-type (*wt*) and eleven F-box mutants (*pof*). Cells of the indicated genotypes were incubated in YES supplemented with 0.1 mg/ml of CHX for the indicated times at 30°C. The anti-FLAG (M2) antibody was used for Western blotting. Tubulin levels were also monitored as a loading control. (B) Relative Pol2-FLAG amounts at 0 and 4 h of CHX treatment shown in *A* were quantified using EZQuant-Gel 2.1. Relative intensity of protein bands at 0 h was set to 1 in each experiment. Relative Pol2-FLAG levels in *skp1-94* cells at 25 and 35°C shown in (Figure 3D) were also quantified.
(EPS)

Figure S4 *swi1*Δ *pof3*Δ cells exhibit replication and chromosome abnormalities. Chromosome samples of *swi1*Δ *pof3*Δ cells were analyzed by PFGE as shown in Figure 5D. The intensities of chromosomes I and II are much lower than that of chromosome III.
(EPS)

Figure S5 Proteasome defect provokes mitotic catastrophes and replication defects in *swi1*Δ cells. (A) Mitotic phenotypes of *wt*, *swi1*Δ, *mts3-1*, and *swi1*Δ *mts3-1* cells with or without genotoxic agents were investigated. Exponentially growing cells were shifted to 30°C for 3 h and fixed in ethanol and stained with DAPI. *swi1*Δ *mts3-1* cells undergo mitotic catastrophes. Quantification of cells with defective chromosome segregation was performed. More than 300 cells were counted at each time point. (B) Representative images of observed nuclear phenotypes in *A* are shown. The scale bar represents 10 µm. (C) DNA damage sensitivity of *swi1*Δ mutant is increased by *mts3-1* mutation. Five-fold serial dilutions of cells were incubated on YES agar medium supplemented with the indicated amounts of HU and CPT for 4 to 5 days at 25°C. (D) The checkpoint-dependent cell elongation phenotype of *swi1*Δ is abolished by *mts3-1* mutation. Cells of the indicated genotypes were incubated on YES agar medium containing 5 mM HU or 5 µM CPT for 2 days at 25°C and photographed. The scale bar represents 10 µm. (E) *mts3-1* mutation exacerbates replication recovery defects of *swi1*Δ mutants. Cells were incubated in the presence of 10 µM CPT for 4 h at 30°C, then washed and returned into fresh medium. Chromosome samples were examined by PFGE. Representative results of repeat experiments are shown. The size of chromosome III (ch.3) varies between *S. pombe* strains due to recombination at rDNA repeats. (F) Quantification of DNA replication recovery shown in *E*. Chromosome band intensity was quantified using EZQuant-Gel 2.1. Average intensities of the three chromosomes are shown. Error bars correspond to standard deviations obtained from the band intensities of the three chromosomes. Relative average intensity of chromosome bands of mid-log-phase cells (Log) was set to 1 in each cell line.
(EPS)

Figure S6 Stability of Pol2 and Mcm subunits in the presence of camptothecin. CPT causes degradation of Pol2 and Mcm4, but not of Mcm6. Thirty minutes before CHX treatment, cells were exposed to 10 µM CPT. Cellular levels of Pol2-Flag, Mcm6-GFP and Mcm4 were determined at the indicated times after CHX treatment. Tubulin levels were also monitored as a loading control. Representative results of repeat experiments are shown.
(EPS)

Table S1 *S. pombe* strains used in this study.
(DOCX)

Table S2 Primers used in this study.
(DOCX)
