# Supplementary material for: Coordinated Degradation of Replisome Components Ensures Genome Stability upon Replication Stress in the Absence of the Replication Fork Protection Complex
Source: PLoS Genet. 2013 Jan 17;9(1):e1003213. doi: 10.1371/journal.pgen.1003213 (PMC3547854; doi:10.1371/journal.pgen.1003213)
Supplement: Table S1 — S. pombe strains used in this study. (DOCX) [file pgen.1003213.s007.docx]

**Supplementary table S1. *S. pombe* trains used in this study**

| Strain | *Genotype* | Reference |
| --- | --- | --- |
| Y1 | *leu1-32 ura4-D18* | lab stock |
| Y256 | *swi1::kan^r^ leu1-32 ura4-D18* | lab stock |
| Y641 | *orc1::5xFLAG-orc1 cdc25-22 leu1-32 ura4-D18* | H. Masukata |
| Y1242 | *pol2-3FLAG:Kan^r^ leu1-32 ura4-D18* | lab stock |
| Y1243 | *pol3-3FLAG:Kan^r^ leu1-32 ura4-D18* | lab stock |
| Y1893 | *mcm2-GFP:hph leu1-32 ura4-D18* | this study |
| Y1895 | *mcm6-GFP:hph leu1-32 ura4-D18* | this study |
| Y1934 | *cdc25-22 pol2-3FLAG:Kan^r^ leu1-32 ura4-D18* | lab stock |
| Y1935 | *cdc25-22 pol3-3FLAG:Kan^r^ leu1-32 ura4-D18* | lab stock |
| Y2750 | *cdc25-22 cdc45*::*FLAG-cdc45:ura4*^+^ | H. Masukata |
| Y2799 | *cdc25-22 cdc45*::*FLAG-cdc45:ura4*^+^ *swi1::hphMX6* | this study |
| Y2804 | *cdc25-22 pol2-3FLAG:Kanr swi1::hphMX6 leu1-32 ura4-D18* | this study |
| Y2806 | *cdc25-22 pol3-3FLAG:Kan^r^ swi1::hphMX6 leu1-32 ura4-D18* | this study |
| Y2837 | *swi1::natMX6 leu1-32 ura4-D18* | this study |
| Y2882 | *mcm2-GFP:hph cdc25-22ts leu1-32 ura4-D18* | this study |
| Y2884 | *mcm6-GFP:hph cdc25-22ts leu1-32 ura4-D18* | this study |
| Y2888 | *mcm6-GFP:hph swi1::kanMX6 cdc25-22ts leu1-32 ura4-D18* | this study |
| Y2890 | *mcm2-GFP:hph swi1::kanMX6 cdc25-22ts leu1-32 ura4-D18* | this study |
| Y2984 | *mts3-1 leu1-32 ura4-D18* | lab stock |
| Y2986 | *mts3-1 mcm6-GFP:hphMX6 leu1-32 ura4-D18* | this study |
| Y2989 | *mts3-1 mcm2-GFP:hphMX6 leu1-32 ura4-D18* | this study |
| Y2990 | *mts3-1 pol3-FLAG:kanMX6 leu1-32 ura4-D18* | this study |
| Y2994 | *mts3-1 pol2-FLAG:kanMX6 erc1-myc:kanMX6 leu1-32 ura4-D18* | this study |
| Y3024 | *pol2-3FLAG:kanMX6 skp1-94 leu1-32 ura4-D18* | this study |
| Y3050 | *swi1::kanMX6 mts3-1 leu1-32 ura4-D18* | this study |
| Y3058 | *swi1::natMX6 mts3-1 mcm6-GFP:hphMX6 leu1-32 ura4-D18* | this study |
| Y3060 | *swi1::natMX6 mcm6-GFP:hphMX6 leu1-32 ura4-D18* | this study |
| Y3062 | *swi1::kanMX6 mts3-1 mcm2-GFP:hphMX6 leu1-32 ura4-D18* | this study |
| Y3064 | *swi1::kanMX6 mcm2-GFP:hphMX6 leu1-32 ura4-D18* | this study |
| Y3070 | *swi1::natMX6 mts3-1 pol3-FLAG:kanMX6 leu1-32 ura4-D18* | this study |
| Y3073 | *swi1::natMX6 pol3-FLAG:kanMX6 leu1-32 ura4-D18* | this study |
| Y3088 | *pol2-FLAG:kanMX6 swi1::natMX6 mts3-1 leu- ura-* | this study |
| Y3090 | *pol2-FLAG:kanMX6 swi1::natMX6 leu- ura-* | this study |
| Y3091 | *pol2-FLAG:kanMX6 swi1::natMX6 leu- ura-* | this study |
| Y3093 | *orc1::5xFLAG-orc1 cdc25-22 swi1::hphMX6 leu1-32 ura4-D18* | this study |
| Y3168 | *pol2-FLAG:kanMX6 swi1::natMX6 skp1-94 leu1-32 ura4-D18* | this study |
| Y3509 | *smt0 pof9::nat Pol2-5FLAG:hphMX6 rpl42-cyhR mat1m-cyhS leu1-32 ura4-D18 ade6-210* | this study |
| Y3510 | *smt0 pof14::nat Pol2-5FLAG:hphMX6 rpl42-cyhR mat1m-cyhS leu1-32 ura4-D18 ade6-210* | this study |
| Y3511 | *smt0 pof5::nat Pol2-5FLAG:hphMX6 rpl42-cyhR mat1m-cyhS leu1-32 ura4-D18 ade6-210* | this study |
| Y3514 | *smt0 pof2::nat Pol2-5FLAG:hphMX6 rpl42-cyhR mat1m-cyhS leu1-32 ura4-D18 ade6-210* | this study |
| Y3526 | *smt0 pof7::nat Pol2-5FLAG:hphMX6 rpl42-cyhR mat1m-cyhS leu1-32 ura4-D18 ade6-210* | this study |
| Y3552 | *smt0 pof3::nat Pol2-5FLAG:hphMX6 rpl42-cyhR mat1m-cyhS leu1-32 ura4-D18 ade6-210* | this study |
| Y3553 | *smt0 pof10::nat Pol2-5FLAG:hphMX6 rpl42-cyhR mat1m-cyhS leu1-32 ura4-D18 ade6-210* | this study |
| Y3622 | *smt0 Pol2-5FLAG:hphMX6 rpl42-cyhR mat1m-cyhS leu1-32 ura4-D18 ade6-210* | this study |
| Y3624 | *smt0 pof4::nat Pol2-5FLAG:hphMX6 rpl42-cyhR mat1m-cyhS leu1-32 ura4-D18 ade6-210* | this study |
| Y3626 | *smt0 pof8::nat Pol2-5FLAG:hphMX6 rpl42-cyhR mat1m-cyhS leu1-32 ura4-D18 ade6-210* | this study |
| Y3628 | *smt0 pof11::nat Pol2-5FLAG:hphMX6 rpl42-cyhR mat1m-cyhS leu1-32 ura4-D18 ade6-210* | this study |
| Y3630 | *smt0 pof13::nat Pol2-5FLAG:hphMX6 rpl42-cyhR mat1m-cyhS leu1-32 ura4-D18 ade6-210* | this study |
| Y3688 | *pof3::ura4MX leu1-32 ura4-D18* | this study |
| Y3694 | *pof3::ura4MX swi1::natMX leu1-32 ura4-D18* | this study |
| Y3684 | *pof3-Myc:hphMX6 pol2-3FLAG:kanMX6 leu1-32 ura4-D18* | this study |
| Y3686 | *pof3-Myc:hphMX6 pol2-3FLAG:kanMX6 swi1::natMX leu1-32 ura4-D18* | this study |
| Y3693 | *pof3::ura4MX swi1::natMX leu1-32 ura4-D18* | this study |
