# Supplementary material for: Coordinated Degradation of Replisome Components Ensures Genome Stability upon Replication Stress in the Absence of the Replication Fork Protection Complex
Source: PLoS Genet. 2013 Jan 17;9(1):e1003213. doi: 10.1371/journal.pgen.1003213 (PMC3547854; doi:10.1371/journal.pgen.1003213)
Supplement: Table S2 — Primers used in this study. (DOCX) [file pgen.1003213.s008.docx]

**Supplementary table S2. Primers used in this study.**

| Gene | Primer | Sequence |
| --- | --- | --- |
| *swi1* | Pswi1-5' | ctg aat gcc att tta atg cg |
|  | Pswi1-3'module | ggg gat ccg tcg acc tgc agc gta cgt caa gtt gat ttg tta aat caa aag c |
|  | swi1CT-5' | cgt cga gaa gcg tta aat aag |
|  | swi1CT-3'module | ggg gat ccg tcg acc tgc agc gta cga atc cga tga aga att ctc gct gag |
|  | Tswi1-5'module | gtt taa acg agc tcg aat tct aat aaa taa aat atc ttc gat tc |
|  | Tswi1-3' | cat gat cct tta gtc gag tg |
| *pof3* | Ppof3-5' | tga aaa gca taa caa ata agt ag |
|  | Ppof3-3'module | ggg gat ccg tcg acc tgc agc gta cgg aat cca aat ctt aat ttc tat acc |
|  | pof3CT-5' | gat tcc tga taa cgt tgc tct tc |
|  | pof3CT-3'module | ggg gat ccg tcg acc tgc agc gta cga tat tag gcg tat ctt ttt tgt ac |
|  | Tpof3-5'module | gtt taa acg agc tcg aat tct aat atg atc ata ttg tct tta tat att c |
|  | Tpof3-3' | cta taa ata agt gtg aat act tac |
| *mcm2* | mcm2CT-5' | cga gac ttt ata gcg ata tg |
|  | mcm2CT-3'module | ggg gat ccg tcg acc tgc agc gta cga aat aag ata ttt agc aaa tgt tct tg |
|  | Tmcm2-5'module | gtt taa acg agc tcg aat tct aat ttt ttg aca caa tca gtt tat gg |
|  | Tmcm2-3' | cct agt atc tat agc gaa tag tc |
| *mcm6* | mcm6CT-5' | gac cta gtt cag tca tat ctc |
|  | mcm6CT-3'module | ggg gat ccg tcg acc tgc agc gta cga gtt cgg aac atc gcc att gct cag |
|  | Tmcm6-5'module | gtt taa acg agc tcg aat tct aag gtt ata aaa gat tcg taa cg |
|  | Tmcm6-3' | ctg ata aat gtc tag atc agg |
